# Subclinical hypothyroidism: association with cardiovascular risk factors and components of metabolic syndrome

**DOI:** 10.1080/13102818.2014.991136

**Published:** 2014-12-16

**Authors:** Milica M. Pesic, Danijela Radojkovic, Slobodan Antic, Radivoj Kocic, Dobrila Stankovic-Djordjevic

**Affiliations:** ^a^ Clinic for Endocrinology, Diabetes and Metabolic Disorders, Clinical Center Nis, University of Nis, Nis, Serbia; ^b^ Department of Microbiology-Virusology, Institute for Public Health Nis, University of Nis, Nis, Serbia

**Keywords:** subclinical hypothyroidism, metabolic syndrome, cardiovascular risk

## Abstract

The aim of this cross-sectional study was to evaluate the cardiovascular risk in patients with subclinical hypothyroidism (SH) and metabolic syndrome (MetS) components. The study included 60 patients with SH and a control group of 60 healthy volunteers, gender and age matched, with normal thyroid-stimulating hormone (TSH) and free thyroxin (FT4) concentration. The following measurements were made in all participants: TSH, FT4, thyroid peroxidase antibodies, anti-thyroglobulin antibodies, body mass index (BMI), waist circumference, blood pressure, fasting plasma glucose, total cholesterol (TC), low-density lipoprotein (LDL) cholesterol, high-density lipoprotein (HDL) cholesterol, triglycerides (TG), TC/HDL cholesterol and LDL/HDL cholesterol ratio, basal insulin level and homeostatic model assessment insulin resistance (HOMA-IR) index. MetS was diagnosed according to the National Cholesterol Education Program Adult Treatment Panel III criteria. The results showed that the following indices were statistically significantly higher in the SH group: BMI (*p* < 0.05), diastolic blood pressure (*p* < 0.001), TC (*p* < 0.05), TG (*p* < 0.05) and basal insulin level (*p* < 0.05). Although MetS parameters were present in a higher per cent in the SH group, there was a significantly higher number of patients with hypertension and decreased HDL cholesterol (*p* < 0.05). More frequently, MetS was diagnosed in SH patients (46.67%) than in the control group (33.33%), although the difference was not statistically significant. These results indicated that the traditional cardiovascular risk factors were more frequently present in SH patients as compared to euthyroid participants. Our results did not confirm significantly higher presence of MetS in SH patients in comparison with euthyroid respondents.

## Introduction

Subclinical hypothyroidism (SH) is defined as elevated serum concentration of thyroid-stimulating hormone (TSH) while the levels of circulating thyroid hormone are within the normal range. The incidence of SH varies between 4% and 10% depending on sex and age. The frequency of this mild thyroid dysfunction increases with age and its frequency is significantly higher in women. SH is reported to occur in 5% of women and 4% of men. In people over the age of 60, the prevalence is significantly increased and reaches 15% in the female and 8% in the male part of the population.[1,2].

According to the TSH level, SH is classified into the following categories: moderately elevated TSH levels (4.0–10.0 mIU/L) and significantly elevated TSH (above 10.0 mIU/L).[3–5] Moderate SH levels are present in the majority (90%) of all patients with SH.

Thyroid dysfunction is associated with dyslipidemia, a well-known cardiovascular risk factor. Besides dyslipidemia, thyroid dysfunction can induce insulin resistance, hypertension, inflammation, oxidative stress, endothelial dysfunction and coagulation disorders, which can also accelerate atherogenesis.

Numerous studies have been conducted to investigate the correlation between SH, acute coronary events and mortality. In a twenty-year follow-up in Whickam Survey, an association between autoimmune thyroid disease and coronary disease was not revealed. However, repeated data analysis involving only the patients with SH showed an increased risk for cardiovascular events and increased mortality in these patients.[6]

Metabolic syndrome (MetS) is defined as a cluster of lipid and non-lipid metabolic factors which increase the risk for cardiovascular disease and/or type 2 diabetes development. In other words, MetS includes insulin resistance, abdominal obesity, dyslipidemia, dysglycemia and hypertension. It is estimated that every fifth person in the world has MetS. The prevalence of MetS in Europe is approximately 15%–35%.[7] However, it is difficult to determine what the real MetS prevalence in the world is because of the variety of MetS definitions used in different studies.[8]

The clinical significance of diagnosing MetS lies in the importance of determining the cardiovascular and metabolic risk (cardiometabolic risk).

Since numerous studies show that increased TSH in overt and SH is a risk factor for accelerated atherogenesis and cardiovascular diseases, there are a growing number of investigations conducted to reveal the possible correlation between SH and MetS. The aim of this cross-sectional study was to evaluate the cardiovascular risk in patients with SH and to compare the presence of MetS components in SH patients with euthyroid ones.

## Subjects and methods

### Subjects

The study group consisted of 60 patients with SH defined as a condition with normal serum levels of free thyroxin (FT4) and elevated serum TSH levels (higher than 4.0 mU/L) repeated in a three-month period. The reference range for FT4 was 10–25 nmol/L, and for TSH, 0.17–4.00 mU/L. Of the patients with SH, 42 (70.00%) were females and 18 (30.00%) males; the average age was 52.00 ± 8.79 years, with a median of 52 years (median was used as a measure of central tendency).

The control group included 60 healthy volunteers, with normal TSH and FT4 levels, gender and age matched: 44 (73.33%) females and 16 (26.67%) males, at an average age of 51.07 ± 10.50 years, with a median of 49.50 years.

None of the included patients had symptoms of hypothyroidism and none of them had ever been on L-thyroxin replacement therapy. The patients had no personal history of thyroid gland surgical treatment, radioactive iodine therapy, neck radiotherapy or treatment with any drug which could provoke thyroid dysfunction (such as amiodarone, lithium, interferon alpha). Other exclusion criteria were: diabetes mellitus, liver lesions, renal dysfunction, congestive heart failure, pregnancy, use of oral contraceptives, statins, steroids and anti-hypertensive therapy. Patients with history of previous cardiovascular events were also excluded.

All the participants gave their informed consent and the study was approved by the institutional ethics committee.

### Indices

TSH and FT4 concentration, thyroid peroxidase antibodies (TPO Ab) and anti-thyroglobulin antibodies (Tg Ab) were measured in all participants. The following measurements were performed in all SH patients: body mass index (BMI), waist circumference, blood pressure, fasting plasma glucose (FPG), total cholesterol (TC), low-density lipoprotein (LDL) cholesterol, high-density lipoprotein (HDL) cholesterol, triglycerides (TG), TC/HDL cholesterol and LDL/HDL cholesterol ratio. Basal insulin level and homeostatic model assessment insulin resistance (HOMA-IR) index were determined in all participants.

MetS was diagnosed according to the modified criteria defined by the National Cholesterol Education Program, Adult Treatment Panel III (NCEP/ATP III) : abdominal obesity defined as waist circumference greater than 102 cm in men and 88 cm in women, hypertriglyceridemia (≥1.7 mmol/L), low HDL cholesterol (in men ≤1.03, and in women ≤1.3), elevated blood pressure (systolic ≥130, and diastolic ≥85 mmHg) and fasting blood glucose over 5.6 mmol/L. Presence of three or more factors was considered sufficient to diagnose MetS.[9,10]

Serum TSH and FT4 concentrations were measured using fluoroimmuno assay (LKB Wallac). Thyroid peroxidase antibodies were determined by coated tube radioimmunoassay (RIA CT) (CIS Biointernationale) and Tg Ab were measured by RIA (Inep Zemun).

### Anthropometric measurements

BMI (kg/m^2^) was calculated as the individual's body mass divided by the square of their height. The waist circumference (cm) was measured midway between the lowest rib and the iliac crest.

### Lipid profile

TC was determined by the enzymatic, colourimetric cholesterol oxidase/peroxidase aminophenazone (CHOD-PAP) methodology, using an enzymatic colour test on a multi-channel analyser Olympus AU 400 (reference value of 3.90–5.50 mmol/L).[11]

TG were measured by the enzymatic, colourimetric glycerophosphate oxidase-peroxidase-4-aminophenazone (GPO-PAP) method, using enzymatic colour test on a multi-channel analyser Olympus AU 400, after enzymatic hydrolysis by lipase (reference value of 0.70–2.0 mmol/L).[12]

HDL cholesterol was measured by a direct immunochemical method on the same device (reference values from 0.86 to 1.5 mmol/L in men, and 1.0 to 1.7 mmol/L in women).[13]

LDL cholesterol was determined using a direct spectrophotometric method (reference value of 2.8–3.90 mmol/L).[14]

TC/HDL cholesterol ratio was calculated as a good predictor of cardiovascular events, and LDL/HDL cholesterol ratio was estimated as an index of atherosclerosis.

### Metabolic parameters

FPG was measured by enzymatic ultra violet test with hexokinase (reference value of 4.2–6.1 mmol/L).[15]

Fasting insulinemia was determined by enzyme-linked immunosorbent assay (ELISA; Biosurse) using a quantitative assay for the measurement. Values were expressed in mU/L.

HOMA-IR index, an insulin resistance indicator, was calculated using the following equation:

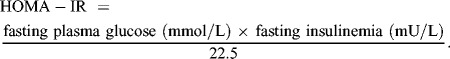



### Blood pressure

Blood pressure was measured in a sitting position after 5 min rest.

### Statistical analysis

Statistical analysis was performed using the SPSS 15.0 package (SPSS Inc., Chicago, IL, USA). Attribute parameters are presented by frequency and percentage. Continuous (measurable) parameters were presented with mean values (X), standard deviation (SD) and median (Md). Chi-square test was used to compare the frequency of the numerical attribute parameters. The Shapiro–Wilk test was used to determine the normality of parameters distribution. Differences were tested by Student's *t*-test for independent samples if the distribution of parameters was normal and Mann–Whitney test was used if the parameters distribution was deviated. The correlation between parameters was determined by Pearson test (*r*), or Spearman test (ρ). The statistical significance of correlation was tested by suitable test (*t-*test for correlation coefficient).

## Results and discussion

Thyroid hormones participate in all aspects of lipid metabolism, including synthesis, mobilization and degradation. The effect which thyroid hormones have on the level of lipid degradation is considered to be a dominant one. Numerous literature data show association between SH and increased TC and LDL cholesterol. One of the possible explanations could be that thyroxin is necessary for gene expression and synthesis of LDL receptors.

### TSH and FT4 levels

In our study, the concentration of TSH was significantly higher in the group of SH patients as compared to that in the age- and gender-matched control group (7.40 ± 1.51 vs. 2.38 ± 0.46 mU/l, respectively; *p* < 0.001). Even though the FT4 level was normal in all patients, the patients with SH had significantly lower FT4 level than that in the control group (12.10 ± 1.15 vs. 14.54 ± 1.06 nmol/l, respectively; *p* < 0.01). A significantly higher number (*p* < 0.001) of SH patients (76.67%) had positive results in the test for thyroid peroxidase antibodies in comparison to the control group (23.33%). Anti-thyroglobulin antibodies were also present in a significantly higher number of patients with SH as compared to the control group (53.33% vs. 20.00%, respectively; *p* < 0.05).

### Anthropometric indices

In the studied SH patients, BMI was statistically significant higher than that observed in the control group (*p* < 0.05). However, the majority of the participants were overweight, albeit not obese. There was no significant difference in the average waist circumference between patients with SH and the control group (Table 1).

**Table 1.** Anthropometric measurements and blood pressure in SH patients and control group.

	SH patients	Control group
Parameters	Mean ± SD Md	Mean ± SD Md
Body mass index (kg/m^2^)	28.0 ± 3.35 (28.00)*	26.37 ± 2.31 (26.00)
Waist circumference (cm)	88.00 ± 11.66 (87.50)	89.20 ± 9.76 (88.00)
Systolic blood pressure (mmHg) (SBP)	130.00 ± 12.66 (130.00)	125.67 ± 8.88 (125.00)
Diastolic blood pressure (mmHg) (DBP)	84.83 ± 10.87 (85.00)^‡^	74.33 ± 8.98 (75.00)

SD – standard deviation; Md – median; *p < 0.05, ^‡^ *p* < 0.001.

### Lipid profile and blood pressure

Considering the presence of cardiovascular risk in SH patients could be meaningful and helpful in making a decision about levothyroxine therapy. The Rotterdam study reported SH to be a strong independent risk factor for myocardial infarction in elderly women.[16] A study in Japan revealed that the risk of myocardial infarction and angina pectoris is doubled in men with SH, comparing to the general population.[17]

In our study, the systolic blood pressure measurements (Table 1) were similar in all participants with no significant difference, while diastolic blood pressure was significantly higher in the studied SH patients as compared to the control group (*p* < 0.001). These results are in agreement with the growing understanding that hypothyroidism is very often a neglected cause of arterial hypertension. The underlying pathophysiological mechanism of hypertension in hypothyroidism possibly involves changes in circulating catecholamine, catecholamine receptors and the renin–angiotensin–aldosterone system. There are reports showing significantly increased diastolic blood pressure in SH patients, comparing to the euthyiroid population.[18,19] One of these studies [18] revealed that 30% SH patients had systolic blood pressure above 140 mmHg in comparison with 16.7% of the euthyroid group. This difference was even more obvious when diastolic blood pressure was compared: 50% SH patients had diastolic blood pressure above 90 mmHg and only 10% of the euthyroid persons. Saltiki et al. [19] showed that in euthyroid individuals the association of thyroid function with diastolic arterial pressure remains significant even when a stricter ‘normal range’ for TSH levels is considered.

Regarding the lipid abnormalities in patients with mild thyroid dysfunction, the literature data differ. There are studies conducted with the aim to evaluate cardiovascular risk in SH that show significantly higher TG in SH patients.[18] On the other hand, some literature data report significant difference between LDL and HDL cholesterol, with minor differences between TC and TG concentrations.[20] Most authors generally agree that SH is accompanied by lipid abnormalities, but there is no consensus about a specific lipid profile characteristic of SH.[21]. Our measurements (Table 2) showed that all lipid status parameters, except for HDL cholesterol, were higher in the studied patients than in the control group. The results obtained by us verified significantly higher values of TC and TG in the group of SH patients as compared to the control group (*p* > 0.05).

**Table 2.** Lipid profile and metabolic parameters in SH patients and control group.

	SH patients	Control group
Parameters	Mean ± SD Md	Mean ± SD Md
Total cholesterol (mmol/l)	5.40 ± 0.62 (5.40)*	5.03 ± 0.19 (5.00)
LDL cholesterol (mmol/l)	3.35 ± 0.44 (3.40)	3.17 ± 0.39 (3.00)
HDL cholesterol (mmol/l)	1.26 ± 0.23 (1.30)	1.45 ± 0.17 (1.50)
Triglycerides (mmol/l)	2.16 ± 0.56 (2.10)*	1.89 ± 0.24 (1.85)
Fasting glycaemia (mmol/l)	4.91 ± 0.97 (4.90)	4.95 ± 1.22 (4.95)
Fasting insulinemia (mU/l)	15.52 ± 1.99 (15.50)*	12.22 ± 2.05 (12.00)
HOMA-IR	2.99 ± 3.0 (3.00)	2.64 ± 2.1 (2.60)

SD – standard deviation; Md – median; *p < 0.05.

### SH and cardiovascular risk factors

The presence of all parameters defined as traditional cardiovascular risk factors (Table 3) was higher in patients with SH. There was a significantly higher (*p* < 0.05) number of patients with blood pressure above 140/90 mmHg and increased TC (≥ 5.2 mmol/L). The number of SH patients with an increased TC/HDL ratio (> 5; *p* < 0.01) and elevated TG (≥ 2.3 mmol/L; *p* < 0.001) were also higher. Triglyceride values of 1.7 and 2.3 mmol/L were used as the TG level intersection point, according to the International Lipid Information Bureau (ILIB) or the regulation of hypertriglyceridemia in adults. In the primary prevention of the cardiovascular events, the desirable TG value is 1.7 mmol/L, whereas TG concentration above 2.3 mmol/L is considered high and requires special attention. Between these two points, the TG values are considered marginal.[22] Although in our study the LDL/HDL ratio was increased in almost 17% of SH patients and only 3.33% of the control group participants had an elevated ratio, the statistical analysis did not find this difference to be significant.

**Table 3.** Traditional cardiovascular risk factors in SH patients and control group.

	SH patients		Control group	
Parameters	*n*	%	*n*	%
Blood pressure ≥ 140/90 mmHg	20	33.33%*	4	6.67%
Total cholesterol (TC) ≥ 5.2 mmol/l	38	63.33%*	18	30.00%
≥ 6.2 mmol/l	8	13.33%	3	5.00%
LDL cholesterol ≥ 3.4 mmol/l	32	53.33%	26	43.33%
≥ 4.1 mmol/l	2	3.33%	1	1.66%
Triglycerides ≥ 1.7 mmol/l	50	83.33%	48	80.00%
≥ 2.3 mmol/l	26	43.33%^‡^	6	10.00%
TC/HDL ratio > 5	16	26.67%^†^	3	5.00%
LDL/HDL ratio > 3.5	19	16.67%	2	3.33%

*p < 0.05, ^†^ *p* < 0.01, ^‡^ *p* < 0.001.

*n* – number of patients.

Some results show that levothyroxine therapy could improve the lipid status in SH patients, especially the TC and LDL cholesterol levels.[23,24] Studies conducted to evaluate the influence of levothyroxine therapy on cardiovascular risk factors and its effects on the early atherosclerosis marker (intima media thickness) revealed improvement in SH patients during levothyroxine therapy.[25,26]

Several reports point to an association between SH and non-traditional cardiovascular risk factors such as increased C-reactive protein concentration (an indicator of low-grade inflammation) hyperhomocysteinemia and possible link between SH and prothrombotic state has also been investigated.[27]

### SH and metabolic syndrome

All MetS parameters (NCEP/ATP III criteria) were present in a higher per cent in the group of SH patients, but only the frequency of hypertension and decreased HDL cholesterol was found to be significantly higher (*p* < 0.05) in SH patients as compared to the control group (Table 4). As a whole, MetS was diagnosed more frequently in SH patients (28 patients, 46.67%) than in the control group (20 patients, 33.33%) but the difference was not statistically significant (*p* > 0.05).

**Table 4.** Metabolic syndrome parameters in SH patients and control group.

	SH patients	Control group
MetS parameters	*n* (%)	*n* (%)
Abdominal obesity	28 (46.67%)	26 (43.33%)
Blood pressure ≥130/≥85 mmHg	22 (36.67%)*	6 (10.00%)
Low HDL cholesterol ‡	38 (63.33%)*	20 (33.33%)
High triglycerides, TG ≥ 1.7 mmol/l	50 (83.33%)	48 (80.00%)
Fasting plasma glucose, FPG ≥ 5.6 mmol/l	8 (13.33%)	6 (10.00%)

*p < 0.05.

‡ Low HDL cholesterol in men ≤ 1.03 mmol/l, in women ≤1.3 mmol/l.

*n* – number of patients.

In a study which analysed 3148 subjects, Garduno-Garcia et al. showed a positive correlation of TSH values with TC, TG, waist circumference, basal insulinemia and HOMA-IR levels. The authors also pointed to an association between FT4 and basal insulinemia. FT4 had an inverse correlation with waist circumference, glycaemia, insulin and HOMA-IR.[28] Velija-Asimi and Karamehic [29] showed that after six months of levothyroxine treatment, patients had normal or limited TSH and, at the same time, the fasting insulin level, the level of fasting and postprandial glucose significantly decreased. Our study showed statistically significantly higher basal insulinemia in the group of patients with SH than in the control group, but there was no significant difference in HOMA-IR (*p* > 0.05) between the two groups (Table 4). Even though there are different cut-off points for HOMA-IR, both groups in our study, showed some degree of insulin resistance.

The majority of the studies conducted in this field report insulin resistance in patients with hypothyroidism. Insulin sensitivity disturbance is usually attributed to decreased intracellular glucose utilization and reduced glucose transporter (GLUT4) translocation. It could be also provoked by decreased glycogen synthesis and reduced glucose oxidation. Similar mechanisms have been shown in SH.[30] As thyroid hormones regulate hepatic glycogenesis, lipogenesis and lipolysis, these hormones play an important role in carbohydrate metabolism. Thyroid hormones can modulate the expression of glucose transporters, adenosine monophosphate-activated protein kinase and acetyl-CoA carboxylase in skeletal muscles.[28] Despite these findings, *in vivo* studies show very different results about insulinemia, insulin resistance and glycaemia in patients with thyroid dysfunction.[27,28,30]

### Correlation between TSH and BMI

The link between TSH levels and body weight is particularly interesting. This relation is complex and could be considered as influence of decreased thyroid function on the body weight, or, conversely, as the impact of abdominal fat on thyroid function mediated by adipocytokines. In the past, there was a general concern that the relationship between TSH and BMI could be modified by smoking habit, but a large study confirmed a positive correlation between TSH and BMI, regardless of smoking.[31] Examining the prevalence of MetS in subclinical and overt hypothyroidism, Erdogan et al. [32] showed a statistically significant difference in BMI between patients with SH and euthyroid subjects. The authors suggested that the effect of mild thyroid failure on the body weight could be crucial for increased cardiovascular risk.

Thus, one of the aims of our study was to examine whether patients with SH have a higher BMI compared to euthyroid persons and our study results confirmed that. When the possible correlation between TSH and BMI, systolic and diastolic blood pressure, lipid profile parameters and metabolic parameters (FPG, fasting insulinemia and HOMA index) was evaluated in the studied SH patients, TSH was found to be significantly correlated with TC and diastolic blood pressure (*p* < 0.05; *r =* 0.37 and *r =* 0.36, respectively). At the same time, it is important to underline that morbid obesity was not present among the participants included in this study, suggesting that increased TSH could not be considered as a consequence of obesity, but rather as a parameter of real mild hypothyroid state.

With growing evidence about association between subclinical/overt hypothyroidism and increased cardiovascular risk, there are numerous studies conducted to evaluate connection between these two different entities, SH and MetS. MetS in patients with thyroid dysfunction could be ‘double jeopardy’ and also explanation for increased cardiovascular morbidity in patients with thyroid diseases. Thus, the results from our study could contribute to the investigations on the correlation between these two seemingly unrelated entities and the factors which associate them.

There are a few studies conducted with the aim to evaluate the association between thyroid dysfunction and MetS.[33,34] Some reports show that even ‘high normal TSH’ is associated with adverse metabolic profile and greater susceptibility to MetS.[35] Garduno-Garcia et al. [28] did not find any significant correlation between SH and MetS defined by the NCEP/ATP III criteria. On the other hand, Erdogan et al. [32] concluded, based on their study results, that MetS was significantly increased in patients with overt hypothyroidism in comparison with SH patients and euthyroid participants. Kota et al. conducted a cross-sectional study to assess thyroid function in patients with MetS diagnosed by NCEP/ATP III and revealed significant positive linear correlation of TSH with TG, TC, LDL cholesterol and significant negative linear correlation with HDL cholesterol. Of the 100 MetS patients participating in their study, 4% had overt hypothyroidism, 22% had SH and 74% were euthyroid. Based on these data, the authors concluded that there was significant association between SH and MetS.[36]

This inconsistency in the literature data about the association between SH and MetS could be due to different diagnostic criteria for MetS, limited number of included patients and/or different general characteristics of the patients.

The results from our study showed significantly increased presence of ‘traditional’ cardiovascular risk factors in the group of patients with SH. These patients had significantly higher BMI, diastolic blood pressure, TC, TG and basal insulin level, as compared to the control group. The frequency of all MetS components defined by NCEP/ATP III criteria, was higher in SH patients than in euthyroid participants, but the difference was statistically significant only in the domain of higher arterial pressure and decreased HDL cholesterol. The smallest difference between the two groups was observed in terms of disglycaemia and abdominal obesity, which is one of the most important components of MetS. However, significantly increased MetS in SH patients, as compared to euthyroid persons, was not confirmed by the results from our study.

It should be noted that our study has some limitations. These are the relatively small number of participants and the cross-sectional study design. Nevertheless, our results add some more to the existing pool of information about the cardiovascular and metabolic risk in SH patients. Naturally, the fact that there might be some association between these two factors does not necessarily indicate a causal relationship. Large prospective randomized control studies are needed in order to provide a more precise picture of this complex issue. Determining whether there is association between SH and cardiovascular risk, or between SH and MetS, may be of clinical importance. Knowledge about the risk degree could be helpful in making the right decision about the necessity of SH treatment.

## Conclusions

The results from our study indicated that traditional cardiovascular risk factors could be more frequent in SH patients as compared to euthyroid participants. However, the statistical analysis showed that the presence of MetS (defined according to the NCEP/ATP III criteria) was not significantly higher in SH patients in comparison with euthyroid participants. Each patient with SH, should be individually evaluated for cardiometabolic risk. Depending on the determined degree of risk, the necessity for replacement therapy should be estimated and a decision about additional measures should be made in order to reduce the cardiovascular and metabolic risk.
